# Spatial-Temporal Variation and Driving Factors of Ecological Vulnerability in Nansi Lake Basin, China

**DOI:** 10.3390/ijerph20032653

**Published:** 2023-02-01

**Authors:** Zhixian Sun, Yang Liu, Hongbin Sang

**Affiliations:** Business School, University of Jinan, Jinan 250002, China

**Keywords:** ecological vulnerability, SRP model, principal component analysis, optimal parameters-based geographical detector, Nansi Lake Basin

## Abstract

Lake basins are one of the most significant areas of human–land interaction. It is essential for the region’s ecological protection and high-quality development to assess their ecological vulnerability (EV) and analyze the key driving factors of EV. Considering the characteristics of the lake basin, we chose 17 indicators to evaluate the EV of the Nansi Lake Basin based on the “sensitivity-resilience-pressure” (SRP) model. Then, spatial principal component analysis (SPCA) and a transfer matrix were used to analyze the spatial-temporal variation characteristics of the EV. Moreover, the optimal parameters-based geographical detector (OPGD) was applied to investigate the factors influencing the spatial heterogeneity of the EV. The results indicated that the EV of the Nansi Lake Basin was characterized by a circling spatial structure, with low values distributed in the Nansi Lake and its surrounding areas, as well as high values concentrated in the northwest. The EV of the Nansi Lake Basin decreased from 2010 to 2020, indicating that the overall ecological pressure in the Nansi Lake Basin decreased. Climatic factors, land use type, and habitat quality were the primary factors that influenced the spatial heterogeneity of the EV in the basin. Our findings can serve as policy guidelines for ecological management and the sustainable development of the Nansi Lake Basin and also contribute to the EV assessment of lake basins.

## 1. Introduction

With the increasing urbanization and deterioration of ecosystem functions, assessing ecological vulnerability (EV) has become one of the most crucial concerns for high economic and social growth as well as sustainable ecological development [1,2]. The connotation of EV, which evolved from the definition of vulnerability, was first introduced into ecology by Clements in the early 20th century [3]. It has since been adopted as a crucial research area by organizations and academic programs around the world, including the International Biological Program (IBP) and the Intergovernmental Panel on Climate Change (IPCC) [4,5]. According to the definition of the concept of EV based on the results of existing studies [6], it can be found that EV is an inherent property determined by its own structure. The EV assessment is often based on a specific spatial area and a specific time scale, and the dynamic changes in the degree of vulnerability of the research object were examined. Overall, the EV reflects the sensitivity of ecosystems to external disturbances and threats and their ability to self-regulate and recover [7,8].

The basins are complex ecosystems with strong interactions between natural processes and human activities [9]. Rapid urbanization and industrial land expansion have neglected the rational planning and protection of the basins’ territorial space, reducing the carrying capacity of resources and the environment [10]. Consequently, an urgent issue of the EV appears in the basins, primarily manifesting in the encroachment and destruction of environmental spaces, severe water pollution, and the continued decline in ecosystem service functions [11,12]. The studies that have been conducted for the Nansi Lake Basin have focused on the assessment of ecosystem service function [13], ecological health assessment [14], landscape pattern evolution analysis [15,16], water environment, and water quality assessment [17,18]. The scope of research is gradually shifting from a single wetland environment to the whole basin. On the issue of EV, Dejun Zhang et al. evaluated the vulnerability of the wetland ecosystem of the Nansi Lake based on the “disturbance-state-sensitivity-development” framework [19]. However, fewer studies have evaluated the EV of the Nansi Lake Basin from the lake basin perspective. Therefore, it is necessary to clarify the spatial distribution characteristics and influencing factors of EV in the Nansi Lake Basin, which can support the formulation of targeted ecological management and restoration strategies.

At present, scholars have extensively evaluated EV at the spatial scales of cities [20,21], counties [22], and river basins [7,23], but studies on the EV in lake basins are still lacking. Unlike river basins, lake basins are characterized by a spatially circling structure, with lakes being the endpoint of the catchment [24]. Meanwhile, lakes are still bodies of water, and their capacity for self-purification is much lower than that of flowing bodies of water such as rivers [25]. With the accumulation of pollutants, the water quality deteriorates severely and endangers the healthy development of basin ecosystems [26]. Notably, climatic factors such as temperature and precipitation are essential drivers of changes in lake and river runoffs [27]. Climate change affects the security of water resources such as groundwater [28], which in turn determines the ecological characteristics of lake basins [24]. Simultaneously, intense and continuous human activities such as agricultural land reclamation and urban expansion have resulted in the deterioration of water quality and ecosystem functions, as well as weakened ecosystem services [29]. In response to the ecological problems in the lake basin, many researchers have focused on ecological risk assessment [30,31] and ecosystem services [9,32]. There is still a need to research the ecological status from the perspective of vulnerability, especially focusing on the impact of climate change and human activities on the EV of lake basins.

The research on EV has been focused on the development of EV assessment systems and data processing models [6]. The determinants of EV at different scales interact with each other and are influenced by the ecological environment, socioeconomic structure, and policies. At present, the EV assessment has changed from context-specific or single-factor assessment models to complex multi-factor assessment models. In the assessment models, EV characterization usually involves terrain conditions, surface conditions, climate change, vegetation conditions, biodiversity degradation, and human activities [33]. The conceptual models for EV assessment mainly include the “sensitivity-resilience-pressure” (SRP) model [1,2], the “exposure-sensitivity-adaptation” (ESA) model [7,34], and the “pressure-state-response” (PSR) model [35]. Among these, the SRP model is based on the connotation of ecosystem stability [36] and has been widely used in EV assessments at the scale of cities and river basins. Nevertheless, the applicability of the SRP model to EV assessment in lake basins still needs to be explored. Concerning the methodology for EV assessment, the emphasis is on determining the weights of the evaluation indicators. The weighting methods mainly include spatial principal component analysis (SPCA) [1,37], entropy method [35,38], analytic hierarchy process (AHP) [39,40], and the fuzzy analytic hierarchy process (FAHP) [23,41]. Among these, the SPCA method can reduce the dimensionality of the data set and effectively solve the problem of covariance among indicators [1,36].

In order to formulate more scientific ecological protection and restoration measures to reduce regional EV, scholars have gradually studied the driving mechanisms of spatial-temporal variations in EV. The geographic detector (GD) is the most commonly used method in existing studies [20,42] because it can effectively detect the spatial heterogeneity of EV [43]. The influencing factor of EV should be the type of quantity in GD models. Continuous-type indicators must be discretized before the model operation, often directly using natural break points [22] and the K-mean classification algorithm [20]. Nevertheless, this artificial division has greater subjectivity. In addition, a few scholars have also used cross-sectional and panel regression models to quantify the effects of environmental and socio-economic factors on the spatial-temporal variability of EV [1]. However, most existing studies were based on county-scale analyses of driving mechanisms, while few scholars were at the grid scale. Different spatial scales have a significant effect on the results [44], and studies are needed based on finer spatial scales to more accurately reveal the influencing mechanisms of the spatial variation in EV for the lake basin.

Therefore, based on the SRP model, our study constructed an index system for the EV assessment of the Nansi Lake Basin from 2010 to 2020. We integrated the SPCA method and transfer matrix to clarify the spatial-temporal evolution characteristics of the EV and further adopted the optimal parameters-based geographical detector (OPGD) model to reveal the driving mechanism of the spatial variation in EV. Our study aimed at verifying the applicability of the SRP model in the EV assessment of the lake basin by fully considering the ecological characteristics of lake basins. Then, we analyzed the spatial and temporal variability of different types of EV, which has been neglected in previous studies of our study area. Compared with previous studies on driving mechanisms, we explored the drivers of EV spatial-temporal variation based on the grid scale. Our study can provide a methodological reference for the EV assessment of lake basins as well as a scientific basis and decision-making reference for the high-quality development and ecological protection of the Nansi Lake Basin.

## 2. Materials and Methods

### 2.1. Study Area

**Figure 1:**
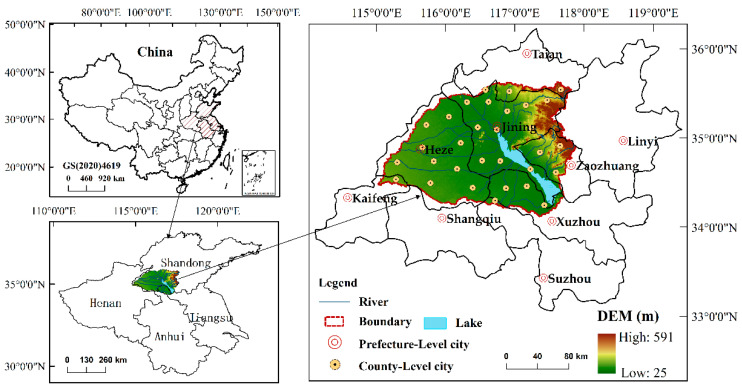
Location of study area.

The Nansi Lake Basin is a typical lake basin, which is located southwest of the Shandong Province at the borders of Jiangsu Province, Anhui Province, and Henan Province (between 34°15′ N to 35°51′ N and 114°57′ E to 117°55′ E). It is an essential agricultural production base in North China, with a total area of approximately 28,300 km^2^ (Figure 1). The basin boasts abundant water resources and multiple tributaries. The Nansi Lake, one of China’s largest freshwater lakes, possesses the features of a wetland environment. It also performs a water transmission and storage function for the east route of the South–North Water Transfer Project [45]. The basin’s geography is mostly flat, and low hills dominate the east area. The annual average temperature is 13–15 °C, and the abundant precipitation is 695.2 mm [46]. The Nansi Lake Basin is a zone with four distinct seasons and abundant biodiversity. In recent years, the ecosystem of the Nansi Lake basin has faced severe threats due to climatic changes and human activities such as resource extraction and urban land expansion [46]. Particularly, the sharp contradiction between humans and land (increasing agricultural production, expanding urban land scale, and long-term disordered development of resources) has led to increasingly severe soil erosion, vegetation damage, and wetlands degradation [39]. These actions greatly restrict the ecological protection and sustainable development of the region. Therefore, it is critical to evaluate the EV of the Nansi Lake Basin to support the healthy and sustainable development of the ecosystem.

### 2.2. Data Source and Processing

The study data primarily consists of topographic data, land use data, soil data, meteorological data, vegetation data, socio-economic data, and boundary data of the Nansi Lake Basin in 2010, 2015, and 2020 (Table 1). The digital elevation model (DEM) data, land use data, GDP data, and population data are from the Resources and Environment Science and Data Center of the Chinese Academy of Sciences (https://www.resdc.cn, accessed on 6 July 2022), with GDP and population data for 2020 replaced by 2019 due to data availability. The soil data are from the National Cryosphere Desert Data Center (http://www.ncdc.ac.cn/portal/, accessed on 2 July 2022) with a spatial resolution of 1 km. The temperature and precipitation data for the basin and its surrounding meteorology were obtained from the China Meteorological Data Service Center (https://data.cma.cn/, accessed on 5 October 2021). The National Ecosystem Science Data Center (http://www.nesdc.org.cn/) provides the normalized difference vegetation index (NDVI) data with a spatial resolution of 30 m. The net primary productivity (NPP) data is from the MOD17A3 data product of the Nation Aeronautics and Space Administration (NASA) (https://ladsweb.modaps.eosdis.nasa.gov/, accessed on 8 July 2022). The Nansi Lake Basin boundary data was obtained from the Nanjing Institute of Geography & Limnology Chinese Academy of Sciences (http://www.niglas.ac.cn/, accessed on 10 May 2021). With the support of the ArcGIS (version 10.8) software platform, all spatial data were unified to a 1 km spatial resolution and the projected coordinate system of Krasovsky 1940 Albers.

### 2.3. Methods

#### 2.3.1. Select Evaluation Indicators

Ecological vulnerability is affected by human activities and natural conditions. The SRP model is a comprehensive evaluation model that takes into account not only the structural characteristics and functions of ecosystems but also the external pressures to which they are subjected. This model covers the components of EV and has been widely used in the study area at different scales, which provides a more comprehensive description of the evolutionary pattern of the EV [20]. Considering the complex environmental characteristics in the Nansi Lake Basin, we selected 17 indicators from ecological sensitivity, resilience, and pressure (Table 2). Thus, the vulnerability evaluation system was developed based on the SRP model according to the results of previous research [1,47]. High-intensity human activities and dramatic climate change have affected lake basins’ runoff and water security, leading to an increasing threat of ecological risk [10,20]. Therefore, climate and human disturbance should be considered essential aspects of the assessment index system.

(1) Ecological sensitivity

Ecological sensitivity reflects the sensitivity of the ecosystems in the basin to external disturbances. It includes terrain conditions, surface conditions, and climatic factors. Elevation, slope, and topographic relief can reflect the topographic conditions that positively impact the EV. Different land use types have varying influences on the EV [1], and they were graded based on previous research findings (Table 3). High soil erosion suggested that the surface was more prone to damage and that the EV was higher [48]. The degree of soil erosion was calculated using the revised universal soil loss equation (RUSLE) [49,50,51] (see Supplementary Materials S1.1). Rainfall erosivity, soil erodibility, slope length, slope gradient factor, vegetation cover factor, and erosion control practice factor should be calculated in the RUSLE. Among them, the erosion control practice factor of the cultivated land was assigned under different slope ranges (see Supplementary Materials, Table S1). Then, the results of the soil erosion were assigned a graded value under the National Soil Erosion Classification and Grading Standard (Table 3). In addition, climatic factors were negative indicators of EV. Areas with high temperatures, abundant precipitation, and high dryness typically had a wetter climate, more vigorous vegetation growth [52,53], richer biodiversity, and lower EV. The average annual temperature and precipitation were obtained by the ordinary kriging interpolation of meteorological station data using ArcGIS (version 10.8) software; based on this, dryness was calculated using the de Martonne method (see Supplementary Materials S1.2).

(2) Ecological resilience

The ability of the ecosystem to recover to its original state under disturbance is called ecological resilience. The NDVI, NPP, landscape diversity and habitat quality are all negative indicators of EV. Because densely forested areas are rich in biological resources, habitat quality is high, and ecosystems are relatively stable [22,53]. The SHDI (Shannon’s diversity index) was calculated by Fragstats (version 4.2) software based on land use data from each period to respond to landscape diversity. The habitat quality index was calculated for the Nansi Lake Basin using the habitat quality module of InVEST (version 3.10.2) software (see Supplementary Materials S1.3), which required the input of threat factor attributes and the sensitivity of habitat types to each threat factor (see Supplementary Materials, Tables S2 and S3). More importantly, the hydrological condition has an important impact on the ecological security of lake basins. We used water yield services and water purification services to characterize hydrological conditions. Among them, water yield services played a key role in improving the hydrological condition of the basin and regulating the regional water cycle [54]. Areas with a low water yield tend to have low vegetation cover, which in turn leads to poor biological survival conditions and lower habitat quality. Water purification services reduce nitrogen and phosphorus nutrients in surface runoff, regulate regional non-point source pollution, and are important indicators of the health of the water environment [9]. The overload of nitrogen and phosphorus output exacerbates the degradation of habitat quality and makes the ecological resilience capacity weak. We measured the water yield and nitrogen and phosphorus output of the Nansi Lake Basin using the InVEST (version 3.10.2) software (see Supplementary Materials S1.4 and S1.5), where the calculation of water purification services requires nitrogen and phosphorus output-related parameter values (see Supplementary Materials, Table S4).

(3) Ecological pressure

Ecological pressure responds to the intensity of disturbance to the ecological environment caused by population and economic activities characterized by population and GDP density. Areas with a high population density and economic development have a higher demand for and exploitation of resources, exacerbating the deterioration of the ecological environment. Hence, population density and GDP density are positive indicators of EV.

#### 2.3.2. Standardization of Indicators

Indicators are distributed at various scales with different units, making them unable to be compared or integrated. Therefore, the range method was applied to standardize the quantitative indicators to a uniform scale, except for the land use type and degree of soil erosion, before calculating the results of the comprehensive EV. To standardize the positive and negative indicators, the following formula can be used:

(1) Positive index

(1) $$Y_{i}=\frac{X_{i}-X_{\min}}{X_{\max}-X_{\min}}$$

(2) Negative index

(2) $$Y_{i}=\frac{X_{\max}-X_{i}}{X_{\max}-X_{\min}}$$

where $Y_{i}$ is the standardized result of the index factor *i*, ranging from 0 to 1, $X_{i}$ is the original data of the index factor *i*, and $X_{\min}$ and $X_{\max}$ represent the minimum and maximum values of the index *i*, respectively.

#### 2.3.3. Spatial Principal Component Analysis

The spatial principal component analysis (SPCA) method is based on the support of the ArcGIS (version 10.8) software to evaluate EV. This method can reduce the dimensions of 17 evaluation indicators and recombine them into mutually unrelated comprehensive indicators. It can effectively avoid the influence of the correlation between the original indicators on the EV assessment. The top *m* principal components with a cumulative contribution rate greater than 85% are extracted to replace the original indicators for analysis. The results are calculated using the following formula:

(3) $$SPCA_{j}=\sum_{i=1}^{n}Y_{i}\times Z_{ij}$$

(4) $$EVI=\sum_{j=1}^{m}SPCA_{j}\times Q_{j}$$

where $SPCA_{j}$ is the value of the principal component *j*, $Y_{i}$ is the standardized value of the original indicator *i* of each principal component, $Z_{ij}$ is the eigenvector of each original indicator *i* of the principal component *j*, *EVI* is the EV index of the study area, $Q_{j}$ is the contribution rate of the principal component *j* and *m* is the number of principal components with a cumulative contribution rate greater than 85%.

#### 2.3.4. The Classification of EV

To make it easier to compare the EV across years and regions, we standardize the results of *EVI* using the range method [20,47], which is calculated as follows:

(5) $$SEVI=\frac{EVI-EVI_{\min}}{EVI_{\max}-EVI_{\min}}$$

where *SEVI* is the standardized value of *EVI*, ranging from 0 to 1, *EVI* is the EV index and $EVI_{\max}$ and $EVI_{\min}$ represent the maximum and minimum values of *EVI*, respectively.

Using the equivalence method, the *SEVI* was classified into five levels based on the previous research findings [20,47]. Level I is slight vulnerability (0 ≤ SEVI<0.2); Level II is mild vulnerability (0.2 ≤ SEVI<0.4); Level III is moderate vulnerability (0.4 ≤SEVI<0.6); Level IV is severe vulnerability (0.6 ≤SEVI<0.8); and Level V is extreme vulnerability (0.8 ≤SEVI ≤1).

In order to compare and analyze the changes in the overall EV of the basin, a multiplier model was introduced to measure the comprehensive EV of the whole basin by year [47], which is as follows:

(6) $$CEVI=\sum_{i=1}^{n}G_{i}\times \frac{A_{i}}{S}$$

where *CEVI* is the comprehensive EV index of the Nansi Lake Basin, $G_{i}$ is the classification level value (I, II, III, IV, V) of *SEVI*, $A_{i}$ is the area of the EV level corresponding to the results of *SEVI*, and S is the total area of the Nansi Lake Basin.

#### 2.3.5. Transfer Matrix

The transfer matrix can be used to analyze the state of the system and its transfer changes at the start and end of the study period. It was introduced to analyze the transfer changes in the various levels of EV in the Nansi Lake Basin through the following equation:

(7) $$S_{ij}=\left[\begin{array}{cccc} S_{11} & S_{12} & \cdots & S_{1n}\\ S_{21} & S_{22} & \cdots & S_{2n}\\ \cdots & \cdots & \cdots & \cdots \\ S_{n1} & S_{n2} & \cdots & S_{nn} \end{array}\right]$$

where *S* stands for the study area, *n* denotes the number of *SEVI* classification categories (n=1, 2, ⋯, 5), and *i* and *j* represent the *SEVI* classification categories at the beginning and end of the study period, respectively.

#### 2.3.6. Optimal Parameters-Based Geographical Detector

The geographical detector model is a technique that is extensively used for spatial stratified heterogeneity analysis [42]. In the traditional GD model, factor detection measures the influence of independent variable X on the spatial heterogeneity of the dependent variable Y in terms of the *q* value; interaction detection measures influence the two–two interactions of different independent variables on the spatial heterogeneity of the dependent variable Y in terms of the *q* value, using the following equation:

(8) $$q=1-\frac{\sum_{h=1}^{L}N_{h}\sigma_{h}^{2}}{N\sigma^{2}}=1-\frac{SSW}{SST}$$

(9) $$SSW=\sum_{h=1}^{L}N_{h}\sigma_{h}^{2},SST=N\sigma^{2}$$

where *q* denotes the explanatory power of a single or interacting independent variable, with larger values of *q* indicating more substantial explanatory power, and *h* denotes the stratification of a single or interacting independent variable or dependent variable. $N_{h}$ and *N* are the numbers of cells in stratum *h* and the whole region, $\sigma_{h}^{2}$ and $\sigma^{2}$ are the variances of the Y values for stratum h and the whole region, respectively. *SSW* and *SST* are the sum of the variances within the stratum and the total variance of the whole region, respectively.

However, spatial data discretization has lacked accurate quantitative assessment in previous studies. An optimal parameters-based geographical detector (OPGD) model was developed to address this issue for a more accurate spatial driving analysis of the driving factors [44]. The OPGD model chooses the set of parameters (discrete method and the number of intervals) with the highest single factor *q* value to determine the best spatial discretization technique for continuous variables. Compared with the GD model, the OPGD model can determine the best spatial data discretization method through quantitative evaluation, which effectively improves the accuracy of spatial data analysis [44]. Meanwhile, the identification of drivers can differ at different spatial scales [55]. Therefore, based on the research results of existing scholars [20,56], we chose the 1 km grid scale to explore the driving mechanisms of EV in the Nansi Lake Basin at different times. With the support of the ArcGIS (version 10.8) software platform, 1 km × 1 km fishing nets were created to generate the fishing net points. Raster data from the *SEVI* and 17 evaluation indicators of the Nansi Lake Basin were extracted to the fishing net points for each period, yielding a total of 27,947 grids that served as the base data for the OPGD model. The parameters at the highest *q* value of the continuous variable were calculated using a combination of discrete methods (quantile method, geometric method, standard deviation method, natural breakpoint method, and equal method) and break numbers (3–6 categories) with the aid of the GD package in the R (version 4.2.1) software. The main influencing factors of EV changes in the Nansi Lake Basin were identified using factor detection and the interaction detection of the OPGD model.

## 3. Results

### 3.1. The Spatial Distribution of EV

The SPCA was used in ArcGIS (version 10.8) software to calculate *EVI* for the years 2010, 2015, and 2020. Specifically, we extracted the top six principal components with a cumulative contribution rate greater than 85% instead of the original indicators to calculate *EVI*. The eigenvalues and contribution rates of principal components are shown in Table 4. After standardizing the data of *EVI*, the *SEVI* was obtained and divided in accordance with the classification criteria in order to better compare and analyze the spatial distribution of EV.

**Figure 2:**
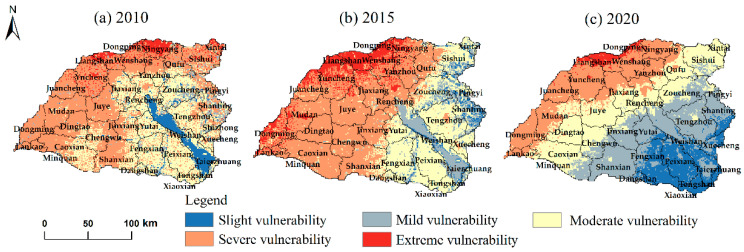
Spatial distribution of standardized ecological vulnerability index in Nansi Lake Basin in 2010 (**a**), 2015 (**b**), and 2020 (**c**).

The spatial distribution of *SEVI* in the Nansi Lake Basin in 2010, 2015, and 2020 is shown in Figure 2. The overall EV showed significant regional differences that increased from the southeast to the northwest and exhibited a circling spatial structure. The ecological conditions in the basin were not good in 2005, mainly in the moderate vulnerability category accounting for approximately 34.93% of the whole basin (Table 5). Furthermore, only 10% or less of the area was slightly and mildly vulnerable, concentrated in Nansi Lake and its surroundings. In 2015, moderate vulnerability decreased by about 6.30% compared to 2010, but the area of severe vulnerability expanded significantly, accounting for 43.69%. Combined with the land use status, we also found that the extreme vulnerability was more scattered in the construction land. In 2020, the percentage of severe vulnerability decreased significantly, only accounting for 2.33%, with severe vulnerability down roughly 22.22% from 2015. In general, the *CEVI* has decreased by 20.48% from 2010 to 2020, indicating that there was a greater improvement in the basin’s ecosystem. It can be seen that the unique ecological restoration and treatment (with Nansi Lake as the core) carried out in recent years has been quite effective and contributed to the improvement of the ecosystem function of Nansi Lake and its surrounding areas. However, the ecological problems in the northwest part of the Nansi Lake Basin cannot be ignored.

### 3.2. Dynamic Changes of EV

**Figure 3:**
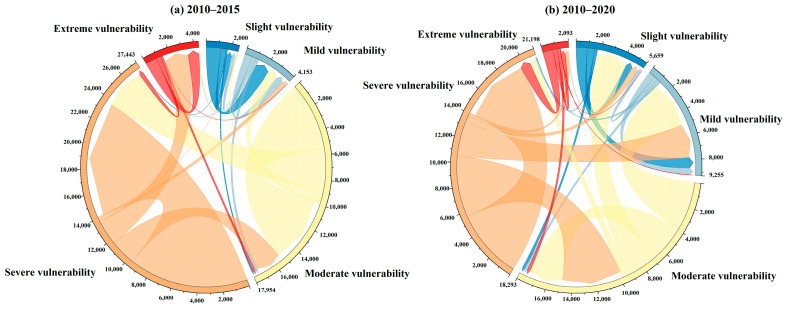
Dynamics change in ecological vulnerability categories for the Nansi Lake Basin during 2010-2015 (**a**) and 2010-2020 (**b**).

In order to better comprehend EV change from 2010 to 2020, we used the ArcGIS (version 10.8) software to calculate the transfer matrix of *SEVI* during the periods 2010–2015 and 2010–2020 (see Supplementary Materials, Tables S5 and S6). The results were visualized using chord diagrams in Origin (version 2022b) software (Figure 3). During 2010–2015, moderate and severe vulnerability dominated the basin. Except for the mutual transfer of the same category, the conversion from moderate vulnerability to severe vulnerability was the largest, covering about 3441 km^2^, implying that the ecological environment of some moderate vulnerability continued to deteriorate. Additionally, the slight and mild vulnerability remained stable. Compared with 2010–2015, the vulnerability levels of 2010–2020 changed significantly. There were 4903 km^2^ regions experiencing the transition from moderate vulnerability to mild vulnerability, followed by a shift from severe vulnerability to moderate vulnerability, indicating that the overall ecological condition of the Nansi Lake Basin improved.

### 3.3. Different Administrative Regions of EV

The mean *SEVI* of counties in the Nansi Lake Basin was extracted from vector data of county-level administrative units using the zoning statistics function of ArcGIS (version 10.8) software (Table 6). Combined with Figure 2, it was clear that counties located on the northwestern were under consistently high vulnerabilities in the ecosystem, while the southeastern cities were greatly reduced.

As shown in Table 6, the mean *SEVI* of Weishan County has always been in the mildly vulnerable zone. For a long time, the county has been committed to ecological restoration, actively carrying out projects such as converting farmland to wetlands and greening barren hills. It also conducted large-scale ecological restoration work for the Nansi Lake, effectively improving its ecological carrying capacity. The EV of the counties surrounding the Nansi Lake, such as Peixian, Fengxian, and Yutai, had been reduced to a mildly vulnerable level under the strengthening of ecological protection and restoration with the Nansi Lake as the core. In contrast, Wenshang, Liangshan, and Yuncheng in the northwest were almost always in the severely vulnerable zone, which means that they had lower ecological resilience to disturbance. Among them, Liangshan had a particularly severe EV problem, with a 0.8543 mean *SEVI* in 2015, placing it in the extremely vulnerable zone. This county was rich in mineral resources, but the over-exploitation of non-coal mineral resources, including single-point limestone mines, seriously damaged the ecological landscape and functions. Meanwhile, the ecological restoration and treatment of the geological environment of mines was expensive and took a long time.

### 3.4. Factors Influencing the Spatial Heterogeneity of EV

#### 3.4.1. Factor Detection Results

The OPGD model was used to investigate the impact of 17 evaluation indicators (X1–X17) on the spatial heterogeneity of *SEVI* in 2010, 2015, and 2020, respectively. We can determine the optimal spatial discretization method for each indicator through the model (Table 7). Among them, X4 (land use type) was spatially discretized according to the land use/cover change (LUCC) classification standard, and X5 (soil erosion degree) was discretized by the professional experience of the National Soil Erosion Classification and Grading Standard. Then, the rest indicators were discretized by the optimal combination of parameters (discrete method and the number of intervals) when maximizing the *q* value. Taking 2015 as an example, using the standard deviation method, the *q* values of X1 (Elevation), when divided into six categories, were greater than other discrete methods.

Table 7 shows that the *q* value of X12 (habitat quality) in 2010 was 0.748, which is more significant than the other variables, indicating that it was the dominant factor affecting the EV in the Nansi Lake Basin that year. The variables X4 (land use type) and X13 (water yield) were followed, with *q* values of 0.746 and 0.537, respectively. In 2015, the top three driving variables (X6–X8) were all climatic factors, which showed a strong impact on the spatial pattern of EV. In 2020, the most influential factors in EV were variables X7 (average annual precipitation), X8 (dryness), and X13 (Water yield). In short, it can be seen that over time, habitat quality, average annual temperature, average annual precipitation, and water yield have become the main drivers of EV spatial heterogeneity. Noteworthily, the influence of climatic factors such as average annual precipitation and dryness has gradually increased.

#### 3.4.2. Interaction Detection Results

**Figure 4:**
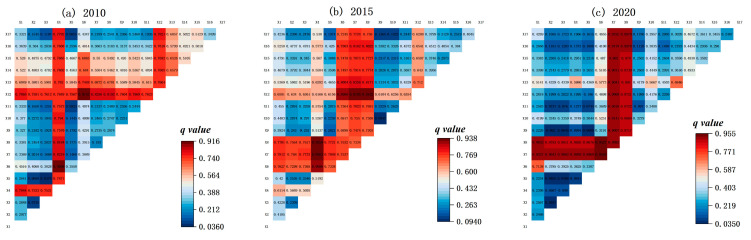
The results of interaction detection of ecological vulnerability in 2010 (**a**), 2015 (**b**) and 2020 (**c**).

In our study, the OPGD model was also used to detect the effects of bivariate interactions on the EV spatial heterogeneity in the Nansi Lake Basin. The interaction results were visualized with the help of heat maps in Origin (version 2022b) software (Figure 4).

From 2010 to 2020, the results of the bivariate interactions were either non-linearly enhanced or bi-factorially enhanced. This means that the interaction of any two variables was more significant than the effect of a single variable on the EV. In 2010, the interaction between variables X12 (habitat quality), X4 (land use type), and other variables significantly influenced the EV spatial heterogeneity. The largest *q* value was the interaction between variable X12 (habitat quality) and X6 (average annual temperature) at 0.914. In 2015, the interaction between variable X12 (habitat quality), X4 (land use type), and climate factors (X6–X8) was greater than 0.85, most likely relating to single-factor detection results. In 2020, the interaction between the variables X6 (average annual temperature) and X7 (average annual precipitation) was the strongest, with a *q* value of 0.955. Moreover, the *q* values of the interactions between X7 (average annual precipitation), X8 (dryness), and other variables were higher than 0.85, indicating that these two variables were significant determinants of EV in that particular year.

In general, the interaction between X12 (habitat quality), X4 (land use type), and climatic factors (X6–X8) better explained the EV spatial heterogeneity. It also suggested that climatic factors such as average annual temperature, average annual precipitation, and dryness were critical drivers of EV in the Nansi Lake Basin.

## 4. Discussion

Green basin governance is a fundamental strategic requirement for promoting high-quality regional development. The Nansi Lake Basin is an essential agricultural production base and green ecological security barrier in Shandong Province. The EV assessment of the basin can provide a scientific reference for ecological governance decisions.

Our study evaluated the EV in the Nansi Lake Basin using the SRP model. The results showed that EV exhibited a circling structure that is consistent with the spatial characteristics of lake basins [24]. The areas with lower EV were mainly concentrated in the Nansi Lake and the low hilly areas to the east, which were usually rich in biodiversity and had high habitat quality [32]. In recent years, governments have continued to strengthen ecological co-protection and management in the Nansi Lake Basin. The provincial government uses Nansi Lake as the core area for ecosystem protection and carries out ecological afforestation and greening along the lake’s shoreline to build ecological corridors. The local governments have implemented a series of water environmental protection projects to improve the ecosystem functions of Nansi Lake. In consequence, ecosystem services such as water storage, water purification, soil conservation, and biodiversity maintenance have significantly enhanced and promoted the ecological security of the Nansi Lake Basin.

Our results showed that Weishan county had the lowest EV, meaning that it is ecologically safer with a higher capacity for ecological recovery and resistance. Similarly, the EV of counties around Nansi Lake has been reduced due to a series of ecological restoration policies for the Nansi Lake Nature Reserve [45]. Conversely, the northwest parts of the basin have higher EV values, indicating lower ecological security. The study of Lv et al. (2012) can indirectly verify this conclusion [57]. In order to improve the whole ecological safety of the basin, ecological protection and restoration policies should be considered and integrated, focusing on systemic integrity and strengthening common protection and joint management. It is necessary to promote the construction of ecological safety corridors in the whole basin and improve the ecosystem functions of the regions, such as water storage and biodiversity maintenance. On the one hand, governments should continue to enhance ecosystem protection with Nansi Lake as the core and carry out ecological afforestation and greening along the lake’s shoreline. On the other hand, more efforts are needed to prevent further ecological deterioration in the northwest regions and increase the effectiveness of land use to lessen the ecological strain of human activities. In addition, it is also necessary to restore the forest landscape of the low hills and green barren slopes in the eastern parts for soil and water conservation.

Climate factors were the dominant factor and became increasingly important over time based on the results of the driving mechanism. Moreover, the Nansi Lake Basin suffered a drought in 2014, which threatened water quality safety, severely damaged the ecological structure and function of the lake, and affected the fisheries industry. Therefore, climate change is a challenging task that must be addressed concurrently with the ecological management of the lake’s basin. The governments should strengthen the monitoring of meteorological indicators as well as ecological changes in the basin. Simultaneously, the cascade effects of climate change in ecological analysis, prediction, and risk warning should be of concern in order to improve the ecological security of the whole basin.

## 5. Conclusions

Based on the SRP model framework, the ecological characteristics of the lake basins were fully considered. There are 17 indicators selected to construct the EV assessment system for the Nansi Lake Basin, which mainly includes the terrain conditions, surface conditions, climatic factors, vegetation conditions, hydrological conditions, and human disturbance. Our study evaluated the spatial-temporal evolution characteristics of EV in the Nansi Lake Basin from 2010 to 2020. Then, we used the OPGD model to detect the drivers of EV spatial heterogeneity. The results showed that the spatial distribution of *SEVI* in the basin was higher in the northwest than in the southeast from 2010 to 2020 and exhibited a circling spatial structure. According to the results of spatial and temporal changes in ecological vulnerability for different categories, we can see that the ecosystem of the basin improved obviously. The EV changes of different county-level administrative units indicated that the mean *SEVI* of southeastern cities generally decreased from 2010 to 2020, primarily distributed around Nansi Lake. In contrast, most cities in the northwest always maintained high values, particularly in the county of Liangshan. Habitat quality, land use type, average annual temperature, average annual precipitation, and water yield were the main drivers of EV spatial heterogeneity. The key roles of climate factors demonstrated a clear growth in strength. Moreover, the EV was jointly influenced by many factors. From the results of interaction factor detection, the strongest degree of interaction was found for climatic factors, land use type, and habitat quality. Therefore, the impacts of land use and climate change on ecological security patterns need to be fully considered in the future environmental management of lake basins. In summary, the findings confirmed the applicability of the SRP model in the EV assessment of the lake basin and also provided support for ecological protection and restoration in the Nansi Lake Basin along with decision-making in the EV assessment of similar lake basins.

## Figures and Tables

**Table 1:** The data used in this study and their sources.

Original Data	Resolution	Source
Digital elevation model (DEM)	90 m	Resources and Environment Science and Data Center (https://www.resdc.cn)
Land use data	1 km	Resources and Environment Science and Data Center (https://www.resdc.cn)
Soil map based Harmonized world Soil Database (v1.2)	1 km	National Cryosphere Desert Data Center (http://www.ncdc.ac.cn/portal/)
Meteorological data	-	China Meteorological Data Service Center (https://data.cma.cn/)
Normalized difference vegetation index (NDVI)	30 m	National Ecosystem Science Data Center (http://www.nesdc.org.cn/)
Net primary productivity (NPP) from MOD17A3	500 m	Nation Aeronautics and Space Administration (NASA) (https://ladsweb.modaps.eosdis.nasa.gov/)
GDP data	1 km	Resources and Environment Science and Data Center (https://www.resdc.cn)
Populations data	1 km	Resources and Environment Science and Data Center (https://www.resdc.cn)
Boundary vector data for the Nansi Lake Basin	-	Nanjing Institute of Geography & Limnology Chinese academy of Sciences (http://www.niglas.ac.cn/)

**Table 2:** The index system of ecological vulnerability assessment in the Nansi Lake Basin.

Target Layer	Criteria Layer	Indicator Layer	Indicator Code	Functional Relationship
Ecological sensitivity	Terrain conditions	Elevation	X1	Positive
		Slope	X2	Positive
		Topographic relief	X3	Positive
	Surface conditions	Land use type	X4	Positive
		Soil erosion degree	X5	Positive
	Climatic factors	Average annual temperature	X6	Negative
		Average annual precipitation	X7	Negative
		Dryness	X8	Negative
Ecological resilience	Vegetation conditions	NDVI	X9	Negative
		NPP	X10	Negative
	Environmental protection	Landscape diversity index	X11	Negative
		Habitat quality index	X12	Negative
	Hydrological condition	Water yield	X13	Negative
		Nitrogen output	X14	Positive
		Phosphorus output	X15	Positive
Ecological pressure	Human disturbance	Population density	X16	Positive
		GDP density	X17	Positive

**Table 3:** Criteria for grading and assigning values to qualitative indicators.

Evaluation Indicators	Standardize Assignments				
	0.2	0.4	0.6	0.8	1
Land use type	Forest land and water body	Grassland	Cultivated land	Construction land	Unused land
Soil erosion grade	Slight	Mild	Moderate	Intense	Extremely intense and violent

**Table 4:** Results of spatial principal component analysis.

Year	Principal Component Coefficients	Principal Components					
		SPCA_1_	SPCA_2_	SPCA_3_	SPCA_4_	SPCA_5_	SPCA_6_
2010	Eigenvalue	0.0608	0.0346	0.0289	0.0265	0.0211	0.0126
	Contribution rate (%)	28.0059	15.9483	13.2982	12.2064	9.7294	5.8256
	Cumulative contribution rate (%)	28.0059	43.9542	57.2524	69.4588	79.1882	85.0138
2015	Eigenvalue	0.1206	0.0526	0.0313	0.0256	0.0131	0.0121
	Contribution rate (%)	40.6916	17.7536	10.5570	8.6413	4.4337	4.0852
	Cumulative contribution rate (%)	40.6916	58.4451	69.0022	77.6435	82.0771	86.1623
2020	Eigenvalue	0.0981	0.0551	0.0430	0.0284	0.0238	0.0140
	Contribution rate (%)	32.3703	18.1596	14.1940	9.3804	7.8544	4.6076
	Cumulative contribution rate (%)	32.3703	50.5299	64.7239	74.1043	81.9587	86.5664

**Table 5:** The results of comprehensive ecological vulnerability index.

Year	Category	Slight	Mild	Moderate	Severe	Extreme	*CEVI*
2010	Area (km^2^)	1705	1168	9868	14,073	1435	3.437
	Area Percentage (%)	6.063	4.135	34.932	49.818	5.079	
2015	Area (km^2^)	842	2985	8087	13,369	2966	3.518
	Area Percentage (%)	2.981	10.567	28.628	47.326	10.498	
2020	Area (km^2^)	3954	8087	8426	7124	658	2.733
	Area Percentage (%)	13.997	28.628	29.828	25.218	2.329	

**Table 6:** Mean standardized ecological vulnerability index in different administrative regions.

County	Mean SEVI			County	Mean SEVI		
	2010	2015	2020		2010	2015	2020
Tongshan	0.4487	0.4164	0.1125	Liangshan	0.7703	0.8543	0.8132
Fengxian	0.4931	0.4575	0.1741	Qufu	0.6303	0.6149	0.5524
Peixian	0.4829	0.4470	0.1625	Zoucheng	0.4810	0.4430	0.3993
Shanting	0.4870	0.2938	0.2471	Ningyang	0.8211	0.7670	0.7107
Tengzhou	0.5414	0.4809	0.2994	Mudan	0.6591	0.7368	0.6257
Rencheng	0.5546	0.7069	0.5368	Dingtao	0.6377	0.6927	0.5277
Yanzhou	0.6331	0.7423	0.6302	Caoxian	0.6209	0.6700	0.4676
Weishan	0.2383	0.3202	0.2460	Shanxian	0.6230	0.6269	0.3058
Yutai	0.5382	0.5020	0.2652	Chengwu	0.6191	0.6500	0.4147
Jinxiang	0.6004	0.6526	0.3634	Juye	0.6449	0.7026	0.5528
Jiaxiang	0.5747	0.7196	0.5877	Yuncheng	0.7408	0.7867	0.7223
Wenshang	0.7222	0.8122	0.7428	Juancheng	0.6659	0.7496	0.6899
Sishui	0.7019	0.4488	0.4582	Dongming	0.6600	0.7839	0.6757

**Table 7:** Spatial discretization methods and factor detection results for each indicator.

Variable Name	2010			2015			2020		
	Discrete Method	Intervals	*q* Value	Discrete Method	Intervals	*q* Value	Discrete Method	Intervals	*q* Value
X1	■	6	0.243	●	6	0.377	■	6	0.197
X2	■	6	0.053	○	6	0.216	○	6	0.032
X3	□	5	0.030	●	6	0.226	□	6	0.035
X4	△	6	0.746	△	6	0.499	△	6	0.083
X5	◆	6	0.025	◆	6	0.174	◆	6	0.029
X6	■	5	0.326	□	6	0.675	■	5	0.271
X7	○	6	0.118	●	6	0.732	●	6	0.897
X8	○	5	0.147	●	6	0.714	●	6	0.867
X9	□	5	0.157	□	6	0.060	○	6	0.036
X10	○	6	0.104	○	6	0.039	○	6	0.267
X11	●	5	0.081	○	6	0.199	○	5	0.067
X12	○	5	0.748	■	6	0.602	■	6	0.184
X13	○	6	0.537	●	6	0.282	●	6	0.408
X14	■	6	0.439	□	6	0.250	●	5	0.241
X15	○	6	0.445	■	6	0.175	■	6	0.205
X16	■	6	0.212	●	6	0.304	●	6	0.090
X17	■	6	0.066	□	5	0.066	■	6	0.150

“△” is by classification criteria, “◆” is by professional experience, “■” represents the quantile method, “□” represents the geometric method, “●” represents the standard deviation method, “○” represents the natural breakpoint method.

## Data Availability

The data that support the findings of this study are available from the corresponding author upon reasonable request and the approval of the data owner.

## Supplementary Materials

The following supporting information can be downloaded at: https://www.mdpi.com/article/10.3390/ijerph20032653/s1. Table S1: P-value of cultivated land under different slope ranges; Table S2: Maximum distance and weights of the threats affecting habitat quality; Table S3: The sensitivity of habitat types for each threat factor; Table S4: Parameter values related to nitrogen and phosphorus output; Table S5: The transfer matrix for different categories of ecological vulnerability from 2010 to 2015; Table S6: The transfer matrix for different categories of ecological vulnerability from 2010 to 2020.
