# Supplementary material for: Spatial-Temporal Variation and Driving Factors of Ecological Vulnerability in Nansi Lake Basin, China
Source: Int J Environ Res Public Health. 2023 Feb 1;20(3):2653. doi: 10.3390/ijerph20032653 (PMC9916223; doi:10.3390/ijerph20032653)
Supplement: Supplementary file 1 [file ijerph-20-02653-s001.zip › ijerph-2024665-supplementary.pdf]

Supplementary Materials

1. The calculation process of relevant indicators

1.1. Soil erosion degree

In this study, we compute the soil erosion in the Nansi Lake Basin using the Revised Universal Soil Loss Equation (RUSLE), which is calculated as follows.

$$A = R \times K \times L \times S \times C \times P \quad \text{(S1)}$$

where A is the amount of soil erosion reduced per unit area, also known as soil conservation; R is the rainfall erosivity factor; K is the soil erodibility factor; LS is the slope length and slope gradient factor; C is the vegetation cover factor; P is the erosion control practice factor.

(1) Rainfall erosivity (R)

The rainfall erosivity is based on rainfall data from several meteorological stations in the Nansi Lake Basin, and the following formula is used to calculate the value of R based on the calculation method of rainfall erosion force factor [53] and the Guidelines for measurement and estimation of soil loss in production and construction projects issued by the Ministry of Water Resources.

$$R_j = 0.0534 P_j^{1.6548} \quad \text{(S2)}$$

where R_j is the annual rainfall erosivity $\left[\mathrm{MJ \bullet mm/(hm^2 \bullet h)}\right]$, P_j is the annual rainfall (mm).

(2) Soil erodibility (K)

The soil erodibility factor is calculated based on the data from the Chinese Soil Dataset (v1.1) based on the Harmonized World Soil Database (HWSD), using the principle of K-value estimation in the EPIC (Erosion Productivity Impact Caculator) model [50,51], with the following equation.

$$\begin{aligned} \mathrm{K} = & \left\{0.2 + 0.3 \exp\left[0.0256 SAN (1 - \frac{SIL}{100})\right]\right\} \times \left[\frac{SIL}{(CLA + SIL)}\right]^{0.3} \\ & \times \left[1 - \frac{0.25 OM}{O + \exp(3.72 - 2.95 OM)}\right] \times \left[1 - \frac{0.7 SN}{SN + \exp(-5.51 + 22.9 SN)}\right] \end{aligned} \quad \text{(S3)}$$

where SAN, SIL, CLA and OM represent the soil sand content (%), silt content (%), clay content (%) and organic carbon content (%), respectively. In addition, $SN = 1 - SAN/100$, and the soil erodibility factor K is converted to the international system multiplied by 0.1317.

(3) Slope length and slope gradient (LS)

Based on the DEM data, the LS value is measured using the topographic relief value [15], which is calculated as follows.

$$LS = H_{\max} - H_{\min} \quad \text{(S4)}$$

where LS is the topographic relief, $H_{\max}$ is the highest elevation value, and $H_{\min}$ is the lowest elevation value.

(4) Vegetation cover factor (C)

The vegetation cover factor plays an important role in soil and water conservation, and the algorithm proposed by Cai et al. through experiments with artificial and natural rainfall is used to estimate the vegetation cover factor [50], calculated as follows.

$$F = \frac{NDVI - NDVI_{\min}}{NDVI_{\max} - NDVI_{\min}} \quad \text{(S5)}$$

$$C=\begin{cases}1 & 0\le F<0.096\\ 0.6508-0.3436\lg(F) & 0.096\le F<0.783\\ 0 & F\ge 0.783\end{cases}\tag{S6}$$

Where C is the vegetation cover factor, F is the degree of vegetation cover, $NDVI_{max}$ and $NDVI_{min}$ represent the maximum and minimum values of normalized vegetation index (NDVI), respectively.

(5) The erosion control practice factor (P)

The erosion control practice factor measures for construction land and water body are assigned a value of 0, while those for unused land, forest land, and grassland were assigned a value of 1 [51]. The slope range was used to assign a value to cultivated land's erosion control practice factor (Table S1).

Table S1. P-value of cultivated land under different slope ranges.

Slope range	0 °- 5 °	5 °- 10 °	10 °- 15 °	15 °- 20 °	20 °- 25 °	≥25 °
P	0.100	0.221	0.305	0.575	0.705	0.800

1.2. Dryness

In this study, the de Martonne method is used to calculate the dryness using two climatic factors, temperature and precipitation, which are calculated as follows.

$$I_{dm}=\frac{P}{T+10}\tag{S7}$$

where I_{dm} is de Martonne dryness, P is the average annual precipitation (mm) and T is the average annual temperature value (°C). Higher values of dryness indicate a wetter climate, rivers with year-round water, constant flow, and sufficient water, and a forested vegetation type [52].

1.3. Habitat quality

We used the Habitat Quality module of the InVEST (Integrated Valuation of Ecosystem Services and Trade-offs) model to calculate the habitat quality Index for the Nansi Lake Basin with the following equation.

$$Q_{xj}=H_{xj}\times\left[1\text{-}\left(\frac{D_{xj}^{z}}{D_{xj}^{z}+k^{z}}\right)\right]\tag{S8}$$

where Q_{xj} is the habitat quality of unit x with land use type j, H_{xj} is the habitat suitability of unit x with land use type j, D_{xj} is the habitat degradation of unit x with land use type j, z is the default parameter of the model, k is the half-saturation coefficient, and the value of k is generally taken as 0. 5.

$$D_{xj}=\sum_{r=1}^{R}\sum_{y=1}^{Y_r}\left(W_r/\sum_{r=1}^{R}W_r\right)r_y\times i_{rxy}\times\beta_x\times S_{jr}\tag{S9}$$

$$i_{rxy} = \begin{cases} 1-\left(\dfrac{d_{xy}}{d_{r\max}}\right) & (\textit{if linear}) \\ \exp\left[-\left(\dfrac{2.99}{d_{r\max}}\right)\times d_{xy}\right] & (\textit{if exponential}) \end{cases} \quad (S10)$$

where D_{xj} is the habitat degradation degree of unit *x* with land use type *j*, W_r is the weight of threat factor *r*, r_y represents the stressor value of unit *y*, β_x represents the reachability degree of unit *x*, S_{jr} is the relative sensitivity of habitat quality of each land use type to threat factors, i_{rxy} is the influence of threat factor *r* in unit *y* on unit *x*, *r* is the habitat threat factor, *y* is the raster in threat factor *r*, d_{xy} is the distance between unit *x* and unit *y*, and $d_{r\max}$ is the influence range of threat factor *r*.

Taking into account the conditions of the Nansi Lake Basin and drawing on previous research [46], we chose Cultivated land, urban land, rural residential land, and industrial and traffic land as threat factors, and determined the maximum stress distance and weights of different threat factors (Table S2), as well as the habitat suitability of different habitat types and the sensitivity of different habitat types to threat factors (Table S3).

Table S2. Maximum distance and weight of the threats affecting habitat quality

Threat factor	Maximum distance	Weight	Spatial decay type
Cultivated land	6	0.75	Linear
Urban land	10	1	Exponential
Rural residential land	9	0.9	Exponential
Industrial and traffic land	12	0.95	Exponential

Table S3. The sensitivity of habitat types to each threat factor.

LUCC code	Land-use type	Habitat suitability	Cultivated land	Urban land	Rural residential land	Industrial and traffic land
11	Paddy field	0.25	0.3	0.5	0.4	0.55
12	Dry land	0.15	0.3	0.5	0.4	0.55
21	Forestland	0.95	0.8	0.9	0.85	0.95
22	Shrubland	0.6	0.55	0.6	0.6	0.7
23	Sparse woodland	0.5	0.5	0.5	0.55	0.65
24	Other woodland	0.4	0.4	0.5	0.45	0.55
31	High coverage grassland	0.8	0.4	0.5	0.45	0.55
32	Medium coverage grassland	0.5	0.45	0.4	0.35	0.45
33	Low coverage grassland	0.3	0.5	0.3	0.25	0.35
41	River canal	0.9	0.75	0.9	0.85	0.95
42	Lake	0.9	0.7	0.9	0.85	0.95
43	Reservoir and pond	0.9	0.7	0.9	0.85	0.95
46	Tidal flat	0.9	0.7	0.7	0.65	0.75
51	Urban land	0	0	0	0	0
52	Rural residential land	0	0	0	0	0
53	Industrial and traffic land	0	0	0	0	0
61	sandy land	0	0	0	0	0
64	Marshland	1	0.75	0.7	0.65	0.75
63	Saline land	0	0	0	0	0
66	Bare rock	0	0	0	0	0
67	Others	0	0	0	0	0

1.4 Water yield

The water yield module of the InVEST model was used to simulate the water yield in the Nansi Lake Basin, which is based on the assumption of the Budyko water-heat coupling equilibrium relationship with the following equation:

$$Y(x) = \left(1 - \frac{AET(x)}{P(x)}\right) \times P(x) \tag{S11}$$

where *Y(x)* is the annual water production of grid cell *x*, *AET(x)* is the annual actual evapotranspiration of grid cell *x*, and *P(x)* is the annual precipitation of grid cell *x*. Based on Budyko's assumption of coupled hydrothermal equilibrium, *AET(x)/P(x)* can be calculated using the improved method of:

$$\frac{AET(x)}{P(x)}=\frac{1+\omega(x)+R(x)}{1+\omega(x)\bullet R(x)+1/R(x)} \quad \text{(S12)}$$

$$\omega(x)=Z\bullet\frac{PAWC(x)}{P(x)} \quad \text{(S13)}$$

$$R(x)=\frac{k(x)\bullet ET_0}{P(x)} \quad \text{(S14)}$$

where $\omega(x)$ is the ratio of modified annual vegetation water availability to expected precipitation for raster cell *x*; *R(x)* is the Budyko drying index of raster cell *x*, implying the ratio of potential evapotranspiration law to precipitation; *k(x)* is the plant evapotranspiration coefficient; ET_0 is the reference crop evapotranspiration, which can be calculated based on methods such as Penman's formula; Z is the Zhang coefficient, which is an empirical constant representing seasonal effects, drawing on the study of Sun Xiaoyin et al., when Z is taken as 29.4 [32], the simulated value of water yield has the smallest error with the actual water yield; *PAWC* is the plant available water content, which can be calculated based on soil texture and organic matter content with the following equation:

$$\begin{aligned} PAWC = {} & 54.509-0.132\times SAN-0.03\times(SAN)^2 \\ & -0.55\times SILT-0.006\times(SILT)^2-0.738 \\ & \times CLAY+0.007\times(CLAY)^2-2.668\times OM \\ & +0.501\times(OM)^2 \end{aligned} \quad \text{(S15)}$$

where SAN, SIL, CLA and O*M* represent the soil sand content (%), silt content (%), clay content (%) and organic carbon content (%), respectively.

1.5 Water purification services

The water purification (Nutrient Delivery Ratio) module of the InVEST model was used to calculate the total phosphorus output and the total nitrogen output of the terrestrial ecological units in the Nansi Lake Basin, which is based on the function that vegetation and soil can convert and store nitrogen and phosphorus pollutants in runoff to achieve water purification, calculated as follows:

$$ALV_x = HSS_x \times pol_x \quad \text{(S16)}$$

$$HHS_x=\frac{\lambda_x}{\lambda_w} \quad \text{(S17)}$$

$$\lambda_x=\log\left(\sum_{u=1}^{U} Y_u\right) \quad \text{(S18)}$$

where ALV_x is the corrected nitrogen and phosphorus output of grid x, HSS_x is the hydrological sensitivity score of grid x, pol_x is the output coefficient of grid x, λ_x is the runoff coefficient of grid x, λ_w is the average runoff coefficient of the study area, and Y_u is the total water yield of grid x and all upstream grids, which can be obtained from the results of the water yield model. The

biophysical properties and nitrogen and phosphorus output coefficients required for the model calculations were determined by referring to the research results [9,13] and the InVEST model guidebook, and the relevant parameters are shown in Table S4.

Table S4. Parameter values related to nitrogen and phosphorus output

Code	Land Use Type	N output coefficient (kg/hm^2)	N filtration rate	Maximum distance of stagnant nutrients (m)	P output coefficient (kg/hm^2)	P filtration rate	Maximum distance of stagnant nutrients (m)
1	Paddy field	19.4	0.3	30	1.22	0.3	30
2	Dry land	14.7	0.4	50	0.59	0.4	50
3	Forestland	2.12	0.8	300	0.15	0.8	300
4	Shrubland	2.12	0.7	260	0.15	0.7	260
5	Sparse woodland	3.15	0.6	250	0.18	0.6	250
6	Other woodland	8.5	0.5	250	2.2	0.5	250
7	High coverage grassland	3.2	0.48	150	0.2	0.4	150
8	Medium coverage grassland	3	0.45	140	0.18	0.38	140
9	Low coverage grassland	2.8	0.4	130	0.16	0.35	130
10	rivers and canals	0.001	0.05	20	0.001	0.05	20
11	Lakes	0.001	0.4	50	0.001	0.4	50
12	Reservoir and pond	0.001	0.05	20	0.001	0.05	20
13	Tidal flat	2	0.5	40	0.05	0.5	40
14	Urban land	12	0.05	10	2.1	0.05	10
15	Rural residential land	20	0.1	10	2.5	0.05	10
16	Other construction land	6	0	10	0.5	0	10
17	Unused land	1.45	0.05	20	0.045	0.05	20

2. Transfer matrix for different categories of ecological vulnerability

2.1. Transfer matrix from 2010 to 2015

In order to better comprehend EV change from 2010 to 2015, we used the ArcGIS (version 10.8) software to calculate the transfer matrix of *SEVI* during the periods 2010–2015 (Table S5).

Table S5. The Transfer matrix from 2010 to 2015

2010\2015	Slight vulnerability	Mild vulnerability	Moderate vulnerability	Severe vulnerability	Extreme vulnerability
Slight vulnerability	323	419	100	0	0

Mild vulnerability	829	1602	545	9	0
Moderate vulnerability	2795	3557	1561	161	12
Severe vulnerability	7	2509	6182	4629	42
Extreme vulnerability	0	0	38	2324	604

2.2. Transfer matrix from 2010 to 2020

In order to better comprehend EV change from 2010 to 2015, we used the ArcGIS (version 10.8) software to calculate the transfer matrix of *SEVI* during the periods 2010–2020 (Table S6).

Table S6. The Transfer matrix from 2010 to 2020

2010\2020	Slight vulnerability	Mild vulnerability	Moderate vulnerability	Severe vulnerability	Extreme vulnerability
Slight vulnerability	565	897	220	23	0
Mild vulnerability	282	538	225	108	15
Moderate vulnerability	2602	3857	2840	565	3
Severe vulnerability	500	2773	4903	5596	302
Extreme vulnerability	5	22	238	832	338
